# Effect of Topical Application of Antimicrobial Peptide PL‐5 (Peceleganan) Spray on Mild to Moderate Infection of Diabetic Foot Ulcers: A Multicenter, Randomized, Double‐Blinded, Placebo‐Controlled Clinical Trial

**DOI:** 10.1111/1753-0407.70255

**Published:** 2026-07-27

**Authors:** Li Qian, Xinjuan Sun, Rourou Chen, Qiuyu Lin, Chen Yu, Guofeng Wang, Wen Hu, Jianfang Che, Aiping Wang, Yuxin Chen, Yu Liu

**Affiliations:** ^1^ Department of Endocrinology Sir Run Run Hospital, Nanjing Medical University Nanjing China; ^2^ Department of Endocrinology JunXie Hospital Nanjing China; ^3^ Department of Orthopedics Sir Run Run Hospital, Nanjing Medical University Nanjing China; ^4^ Department of Endocrinology The First People's Hospital of Lianyungang Lianyungang China; ^5^ Department of Endocrinology Huai'an Second People's Hospital Huai'an China; ^6^ Jiangsu Protelight Pharmaceutical Company Jiangyin China

**Keywords:** antimicrobial peptide, clinical efficacy, diabetes foot ulcers, local infection, PL‐5 spray

## Abstract

**Aims:**

To evaluate the clinical efficacy and safety of topical antimicrobial peptide PL‐5 (Peceleganan) spray combined with standard debridement in the treatment of mildly to moderately infected diabetic foot ulcers (DFUs).

**Materials and Methods:**

This multicenter, randomized, double‐blind, placebo‐controlled trial was conducted in four tertiary hospitals. Eligible patients were randomized to PL‐5 spray or placebo, both receiving standardized debridement. The primary endpoint was clinical response rate at 1 day after end of treatment (EOT1).

**Results:**

Baseline characteristics were balanced between groups. The clinical response rate at EOT1 was significantly higher in the PL‐5 group than in the placebo group (*p* < 0.05). The clearance rate of drug‐resistant bacteria at EOT1 was 71.43% in the PL‐5 group versus 50% in the control group (*p* < 0.05). No significant differences were observed in overall microbiological eradication rates at EOT1 (57.89% vs. 33.33%) or EOT7 (64.71% vs. 40.00%) (both *p* > 0.05). Adverse event rates were 24.24% in the PL‐5 group and 33.33% in the placebo group (*p* > 0.05), with no serious drug‐related adverse events. PL‐5 showed a significant improvement in clinical effective rate, but overall microbiological eradication was not significantly different between groups. However, in the subset of patients with drug‐resistant bacteria, PL‐5 demonstrated a significantly higher clearance rate, suggesting potential for treatment of resistant infections.

**Conclusions:**

Topical PL‐5 spray improves clinical outcomes in mild‐to‐moderate infected diabetic foot ulcers and achieves superior pathogen clearance among drug‐resistant infection subgroups. It represents a promising low‐resistance topical option for managing mild to moderate DFU infections.

**Trial Registration:** Chinese Clinical Trial Registry Identifier: ChiCTR2300068632

## Highlights

Topical PL‐5 spray achieved significantly higher clinical effective rates than placebo in mild to moderate diabetic foot ulcer infections. PL‐5 demonstrated superior clearance of drug‐resistant bacteria (71.4% vs. 50.0%). The treatment showed favorable safety profile and promoted long‐term wound healing.

## Graphical abstract

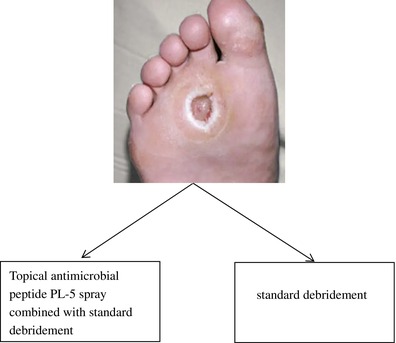

## 1 Introduction

Diabetic foot ulcers are one of the most serious and common chronic complications of diabetes mellitus, with a global incidence of approximately 18.6 million new cases each year [1]. Among these patients, up to 50% of diabetic foot ulcers are complicated with local infection, and as the infection progresses, it can lead to a series of adverse outcomes such as deterioration of the patient's general condition, lower extremity amputation, and even death [2, 3, 4]. Therefore, the rational selection of antimicrobial therapy and the implementation of meticulous wound care are the core links in the clinical management of diabetic foot infections (DFIs), which are directly related to the prognosis of patients.

At present, systemic antibiotic therapy is the main clinical treatment for DFIs, and many clinical studies [5, 6, 7, 8] have confirmed the certain efficacy of various systemic antibiotics in the treatment of DFIs. However, the long‐term and irrational use of systemic antibiotics has led to an increasingly serious problem of bacterial drug resistance, and the potential systemic adverse reactions such as gastrointestinal discomfort, liver and kidney function damage, and disturbance of intestinal flora have also become important factors restricting their clinical application. In contrast, topical antimicrobial agents have unique clinical advantages: they can directly act on the infected wound site, achieve a high local drug concentration, effectively exert antibacterial effects, and at the same time reduce the systemic absorption of drugs, thereby significantly lowering the risk of systemic adverse reactions and the induction of bacterial drug resistance. Therefore, topical antimicrobial agents have become an important choice for the clinical treatment of mild to moderate diabetic foot ulcer infections. Nevertheless, the types of conventional topical antimicrobial agents are relatively limited, and the problem of drug resistance is also gradually emerging, so it is urgent to develop new and efficient topical antimicrobial drugs with novel antibacterial mechanisms.

Antimicrobial peptides (AMPs) are a class of small molecular peptides with broad‐spectrum antibacterial activity that widely exist in organisms, and their unique antibacterial mechanism makes them not easy to induce bacterial drug resistance, becoming a research hotspot in the field of new antimicrobial drugs in recent years. PL‐5 (Peceleganan) spray is a novel topical AMP preparation independently developed for the treatment of skin and soft tissue wound infections. It exerts antibacterial effects by nonspecifically interacting with the cell membrane of pathogenic bacteria, destroying the integrity of the bacterial cell membrane and leading to bacterial death [9, 10, 11]. This unique membrane‐targeting antibacterial mechanism fundamentally avoids the common drug resistance mechanisms of traditional antibiotics such as target gene mutation and drug efflux pump enhancement, and has a good application prospect in the treatment of drug‐resistant bacterial infections.

Previous preclinical and phase IIb clinical studies [12, 13] have confirmed that PL‐5 spray has strong and broad‐spectrum antibacterial activity against both Gram‐positive and Gram‐negative bacteria, and its safety and tolerability are significantly better than traditional topical antibiotics such as silver sulfadiazine cream. The phase IIb clinical trial [13] has further verified that PL‐5 spray is safe and effective in the treatment of skin wound infections, including Wagner grade 2 superficial infected diabetic foot ulcers. Based on the above research foundation, this study further designed a multicenter, randomized, double‐blind, placebo‐controlled clinical trial to systematically evaluate the clinical efficacy and safety of 2‰ concentration PL‐5 spray combined with standard debridement in the treatment of mild to moderate diabetic foot ulcer infections, and to provide high‐level clinical evidence for its clinical promotion and application.

## 2 Patients and Methods

### 2.1 Study Population

This study enrolled adult patients aged 18 to 75 years old, of both genders, who were diagnosed with type 1 or type 2 diabetes mellitus in accordance with the diagnostic criteria of the American Diabetes Association (ADA). Its use in previous DFU infection trials and alignment with Chinese FDA guidelines for topical antimicrobial agents. We have also provided a clear definition of “clinical effective rate” as the proportion of patients achieving a ≥ 50% reduction in wound infection score (based on a composite of erythema, edema, exudate, and pain) at the end of treatment. The inclusion criteria were as follows: (1) suffering from mild to moderate diabetic foot ulcer infection (IWGDF grade 2) diagnosed by the International Working Group on the Diabetic Foot (IWGDF) [14]; (2) glycated hemoglobin (HbA1c) ≤ 10.5% at enrollment, indicating relatively stable blood glucose control; (3) the total score of diabetic foot ulcer wound infection score and wound measurement score did not exceed 15 points (see Table 1 for detailed scoring criteria). Among them, IWGDF grade 2 diabetic foot ulcer infection was defined as the presence of at least two of the following local inflammatory signs: local edema or induration, erythema with a diameter of 0.5–2 cm, local pain or tenderness, skin temperature elevation, and abnormal exudate (thick exudate, clear to white exudate, or hemorrhagic exudate).

**TABLE 1:** Items comprising the diabetic foot infection (DFI) wound score wound parameters and wound measurements and the method for scoring each.

Item	Assessment	Preassigned scoring
Wound parameters		
Purulent discharge	Absent	0
	Present	3
Other signs and symptoms of inflammation^a^	Absent	0
Nonpurulent discharge	Mild	1
Erythema	Moderate^b^	2^b^
Induration	Severe	3
Tenderness		
Pain		
Local warmth		
Range of wound parameter (10‐item) subtotal		0–21
Range of wound parameter (8‐item) subtotal		0–15
Wound measurements		
Size (cm^2^)	< 1	0
	1–2	1
	> 2–5	3
	> 5–10	6
	> 10–30^b^	8^b^
	> 30	10
Depth (mm)		
	< 5	0
	5–9	3
	10–20^b^	7^b^
	> 20	10
Undermining (mm)	< 2	3
	2–5^b^	5^b^
	> 5	8
Range of wound measurement subtotal		3–28
Range of total 10‐item DFI wound score		3–49
Range of total 8‐item DFI wound score		3–43

^a^ Each assessed and placed in one of the pre‐assigned categories.

^b^ For the analysis of the SIDESTEP study data presented here, because there were few patients in the most severe group, some moderate scoring values were combined with the severe group. Wound measurements: Size (cm^2^), the ulcer area as traced with a fine‐tipped felt pen on a sterile clear plastic film applied over the wound. Scored as shown (0–10) based on size of the ulcer (0 to > 30 cm^2^). Depth (mm), measured in the deepest apparent part of the wound using a sterile cotton‐tipped wooden swab held 901 to the wound and marked with a pen held parallel to the surface of the intact skin. Undermining (mm), measurement of any tunneling, subepithelial tissue loss, or shearing, as measured using a sterile cotton‐tipped wooden swab; scored as shown (3–8) based on the measured amount (< 2 mm to > 5 mm). Wound parameters: Purulent drainage, a viscous, yellowish‐white or greenish fluid formed in infected tissue. *Nonpurulent drainage*, a serous, sanguineous, or serosanguineous collection of fluid in the tissue surrounding the wound. Grade: absent: none present; mild: scant drainage noted on dressing (< 0.5 cm diameter) not requiring additional dressings; moderate: greater than scant but less than copious drainage on dressing (> 2 cm diameter) requiring additional dressing changes; severe: copious drainage on dressing (> 2 cm diameter) requiring additional dressing changes. *Erythema*, congestive or exudative redness surrounding the wound caused by engorgement of the capillaries in the lower layers of the skin. Grade: none: absent; mild: pink, barely perceptible; moderate: pale red with defined edges; severe/extreme: red to dark red. *Induration*, inflammatory hardening or thickening of tissues; sometimes called “brawny edema.” Grade: none: absent; mild: localized to the site of infection; moderate: limited extension from the site of infection; severe: extending from the site of infection to involve a substantial portion of the affected lower extremity. *Tenderness* (sign), palpation of the site of infection elicits a report by the patient of tenderness; measured on a 0 (no tenderness) to 10 (the worst imaginable tenderness) scale. Grade: none: absent; mild: score of ≤ 5; moderate: score of 6–8; severe: score of ≥ 9. *Pain* (symptom), subjective reporting of discomfort or the perception of pain at the site of the infection as reported by the patient; measured on a 0 (no pain) to 10 (the worst imaginable pain) scale. Grade: none: absent; mild: score of ≤ 5; Moderate: score of 6–8; Severe: score of ≥ 9. *Local warmth* (sign), Increase in skin temperature relative to the uninfected contralateral foot. Grade: none: temperature of the two sides the same; mild: temperature slightly, but perceptibly warmer; moderate: temperature clearly warmer (perceived as 1°–2°F); severe: marked difference in temperature (perceived as > 2°F).

The exclusion criteria were strictly formulated to ensure the homogeneity of the study population and avoid confounding factors: (1) having multiple ulcer wounds on the lower extremities; (2) accompanied by fever (axillary temperature ≥ 38.5°C) or other signs of systemic infection such as chills, hypotension, and elevated inflammatory indicators; (3) plain X‐ray or other imaging examinations suggesting osteomyelitis of the affected limb; (4) complicated with severe peripheral vascular disease that requires surgical intervention such as vascular bypass or angioplasty; (5) having used systemic or topical antibiotics within 72 h before enrollment; (6) suffering from systemic rheumatic immune diseases that require long‐term use of immunosuppressive drugs or glucocorticoids; (7) having a history of allergy to the main components or excipients of PL‐5 spray; (8) pregnant or lactating women; and (9) patients with severe liver and kidney function damage, malignant tumors and other serious underlying diseases that affect the trial evaluation.

All eligible patients signed an informed consent form voluntarily after being fully informed of the study purpose, trial process, potential benefits and risks. The study was conducted in strict accordance with the Declaration of Helsinki (2013 revision) and the relevant provisions of the Good Clinical Practice (GCP). The study protocol was approved by the medical ethics committee of each participating hospital, and the approval numbers were kept on file for inspection.

### 2.2 Study Design and Implementation

This was a multicenter, randomized, double‐blind, placebo‐controlled parallel group clinical trial conducted in four participating hospitals in Jiangsu Province, China. Based on a 30% difference in clinical effective rate between groups (*α* = 0.05, power = 80%), yielding a required sample of 45 patients (30 PL‐5, 15 placebo). The final enrollment of 48 patients met this requirement. A total of 48 patients with mild to moderate diabetic foot ulcer infections who met the inclusion and exclusion criteria were enrolled in the study. The subjects were randomly assigned to the experimental group and the control group at a ratio of 2:1 by the central randomization method with the help of a random number table: 33 cases in the PL‐5 spray intervention group (standard debridement combined with topical 2‰ concentration PL‐5 spray, once a day) and 15 cases in the placebo control group (standard debridement combined with topical placebo spray without active ingredients, once a day).

The planned course of treatment for the study was 14 days, and the treating physician could appropriately extend the treatment course to 28 days according to the actual wound healing and infection control of the patients; if the patient's wound achieved complete epithelialization and healing during the treatment, the treatment could be terminated in advance, and the end of treatment visit was arranged immediately.

The follow‐up process was standardized and unified for all subjects: the baseline visit was completed on the day of enrollment, including collecting general demographic data, medical history, conducting physical examinations, detecting laboratory indicators, evaluating wound conditions and scoring; the routine follow‐up visits were arranged on the 8th day of treatment, 1 day (EOT1) and 7 days (EOT7) after the end of treatment; for patients with an extended treatment course of 28 days, additional follow‐up visits were added on the 15th and 22nd days of treatment. For patients who withdrew from the study early due to various reasons, the last recorded clinical evaluation data and wound bacterial culture results were taken as the end of treatment data for statistical analysis.

The wound treatment operation was standardized and unified in all participating centers: at each follow‐up visit, the clinicians first performed standard debridement of the infected wound according to the unified operating specifications, removing the necrotic tissue, pus and secretions of the wound to keep the wound bed clean; then collected the wound secretion samples for bacterial culture and drug sensitivity test; after that, the PL‐5 spray or placebo spray was evenly sprayed on the entire wound surface and the surrounding 2 cm normal skin according to the grouping; then covered the wound with two layers of sterile gauze soaked with the corresponding spray, and then covered with Vaseline gauze to keep the wound moist, and finally fixed with sterile gauze bandage. The dressing change operation was performed once a day at a fixed time by the specially assigned person in each participating center to ensure the consistency of the treatment operation.

In order to ensure the objectivity and accuracy of the evaluation results, the study adopted a double‐blind design: both the enrolled patients and the clinical evaluators were unaware of the grouping situation; two independent professional evaluators who were not involved in the clinical treatment of the patients were assigned in each participating center, and they were responsible for taking standardized photos of the wound according to the unified shooting specifications (same angle, same light, same scale) at each follow‐up visit, and the wound photos were numbered and coded for blind evaluation. The diabetic foot ulcer wound infection score and wound measurement score were independently completed by two clinicians in each center, and the average value was taken as the final score to reduce the evaluation bias.

### 2.3 Outcome Measures

#### 2.3.1 Primary Efficacy Endpoint

The primary efficacy endpoint of the study was the clinical effective rate at 1 day after the end of treatment (EOT1), which was comprehensively and qualitatively evaluated by the attending physicians combined with the wound infection signs, symptoms, and objective examination results according to the unified evaluation criteria.

#### 2.3.2 Secondary Efficacy Endpoints

The secondary efficacy endpoints included: (1) clinical effective rate at 7 days after the end of treatment (EOT7); (2) microbiological efficacy rate at EOT1 and EOT7; (3) drug‐resistant bacteria clearance rate at EOT1; (4) wound healing rate at EOT1 and EOT7; (5) the change trend of wound infection score, wound area and wound measurement score from baseline to EOT1; and (6) the average treatment course of the two groups.

#### 2.3.3 Safety Evaluation Endpoints

The safety evaluation endpoints included: (1) general vital signs (body temperature, heart rate, respiratory rate, blood pressure) of the subjects during the treatment and follow‐up period; (2) routine laboratory examination indicators (routine blood test, routine urine test, liver function, kidney function, procalcitonin, etc.); (3) special examination indicators (pregnancy test for women of childbearing age); and (4) incidence of adverse events (AEs) and serious adverse events (SAEs) during the trial, including the type, occurrence time, severity, treatment measures, and outcome of AEs, and the causal relationship between AEs and the study drug was evaluated by the investigator according to the five‐level evaluation method (definite, probable, possible, unlikely, and unevaluable).

### 2.4 Evaluation Criteria for Efficacy Endpoints

#### 2.4.1 Clinical Efficacy Evaluation Criteria

At each follow‐up visit after enrollment, the investigator graded the clinical response of the patient's wound infection according to the following five‐level criteria: (1) *Infection cured:* all local inflammatory signs and symptoms of the wound infection completely disappeared, and the wound was in the healing stage; (2) *Infection improved:* most of the local inflammatory signs and symptoms of the wound infection were significantly improved or partially disappeared, and the infection was effectively controlled; (3) *Treatment failed:* more than one local inflammatory sign or symptom of the wound infection was significantly aggravated, or the infection spread, and the wound condition deteriorated; (4) *Unevaluable:* the patient received less than 10 days of treatment, or lost to follow‐up, or the clinical data were incomplete and could not be evaluated; and (5) *Recurrence:* the infection that had been cured or improved showed aggravation of inflammatory signs and symptoms again after a period of time. The clinical effective rate was calculated as the ratio of the number of patients with “infection cured” and “infection improved” to the total number of evaluable patients.

#### 2.4.2 Microbiological Efficacy Evaluation Criteria

Microbiological efficacy evaluation was only performed in patients with positive bacterial culture results at the baseline visit. The bacterial clearance status was evaluated according to the results of wound bacterial culture at follow‐up, and divided into five levels: (1) *Clearance:* all the pathogenic bacteria isolated at baseline were completely eradicated in the follow‐up bacterial culture; (2) *Presumed clearance:* at least one of the multiple pathogenic bacteria isolated at baseline was eradicated, and no new pathogenic bacteria were isolated; for wounds with too small a size to collect bacterial culture samples due to significant healing, it was also judged as “presumed clearance”; (3) *Treatment failure:* all the original pathogenic bacteria isolated at baseline still persisted in the follow‐up bacterial culture, or the bacterial load increased; (4) *Colonization:* new pathogenic bacteria were isolated in the follow‐up bacterial culture, but there were no corresponding clinical inflammatory signs and symptoms, and no additional antimicrobial treatment was required; and (5) *Secondary infection:* new pathogenic bacteria were cultured in the follow‐up, and accompanied by obvious local inflammatory signs and symptoms, requiring additional clinical antimicrobial treatment. The microbiological effective rate was the ratio of the number of patients with “clearance” and “presumed clearance” to the total number of patients with baseline positive bacterial culture and evaluable microbiological results.

#### 2.4.3 Composite Efficacy Evaluation Criteria

Composite efficacy was a comprehensive evaluation of clinical efficacy and microbiological efficacy, only for patients with positive bacterial culture at baseline. The composite response was divided into three levels: (1) *Cured:* clinical response was cured or improved, and microbiological response was clearance or presumed clearance; (2) *Ineffective:* clinical response was failure or recurrence, or microbiological response was nonclearance, colonization or secondary infection, or both clinical and microbiological efficacy were ineffective; and (3) *Unevaluable:* clinical response or microbiological response was unevaluable, or both were unevaluable.

#### 2.4.4 Wound Healing Rate Evaluation Criteria

The wound healing rate was independently evaluated by two professional evaluators who were blinded to the grouping according to the standardized wound photos taken at each follow‐up visit. The wound area was measured by the planimetric method, and the wound healing rate was calculated according to the formula: Wound healing rate (%) = (Baseline wound area − Follow‐up wound area)/(Baseline wound area × 100%). The average value of the evaluations of the two evaluators was taken as the final wound healing rate of the patient.

### 2.5 Statistical Analysis Methods

All clinical research data were double entered into the Epidata 3.1 database, and logical check and data cleaning were performed to ensure the accuracy and completeness of the data. The statistical analysis was completed by using SPSS 27.0 statistical software, and the test level was set as *α* = 0.05 (two‐sided test). *p* < 0.05 was considered to have a statistically significant difference.

The statistical analysis population was divided into three groups according to the intention‐to‐treat (ITT) principle and per protocol (PP) principle: (1) *Full analysis set (FAS):* all enrolled patients who received at least one dose of the study drug and had at least one postbaseline efficacy evaluation data, with the last observation carried forward (LOCF) for missing data; (2) *Per protocol set (PPS):* patients who strictly complied with the study protocol, completed the entire treatment and follow‐up process, and had no major protocol violations; and (3) *Safety set (SS):* all enrolled patients who received at least one dose of the study drug and had at least one safety evaluation record.

The statistical methods for different types of data were as follows: (1) *Continuous variables:* expressed as mean ± standard deviation (*x* ± *s*) or median (interquartile range) [*M* (*Q*1, *Q*3)] according to the normality test and variance homogeneity test; the comparison between the two groups adopted independent sample *t*‐test for normally distributed data, and Mann–Whitney *U* test for nonnormally distributed data. (2) *Categorical variables:* expressed as case number and percentage (*n*, %); the comparison between the two groups adopted Pearson chi‐square test, and Fisher's exact probability test was used when the theoretical frequency was less than 5. (3) *Repeated measurement data:* the mixed effects model was used for statistical analysis to adjust the influence of baseline variables, treatment course and other confounding factors on the results.

In this study, the clinical and microbiological efficacy endpoints were mainly analyzed based on the PPS population, and the safety endpoints were analyzed based on the SS population; the FAS population was used for sensitivity analysis to verify the robustness of the study results.

## 3 Results

### 3.1 Study Population and Follow‐Up Status

A total of 60 patients were initially screened for eligibility in this study from April 2023 to October 2023, among which 12 patients were excluded after screening: 10 patients did not meet the inclusion criteria, one patient lost interest in the study and voluntarily withdrew, and one patient could not be followed up due to address change and was unreachable. Finally, 48 patients were successfully randomized and enrolled, including 33 patients in the PL‐5 group and 15 patients in the placebo group.

**FIGURE 1:**
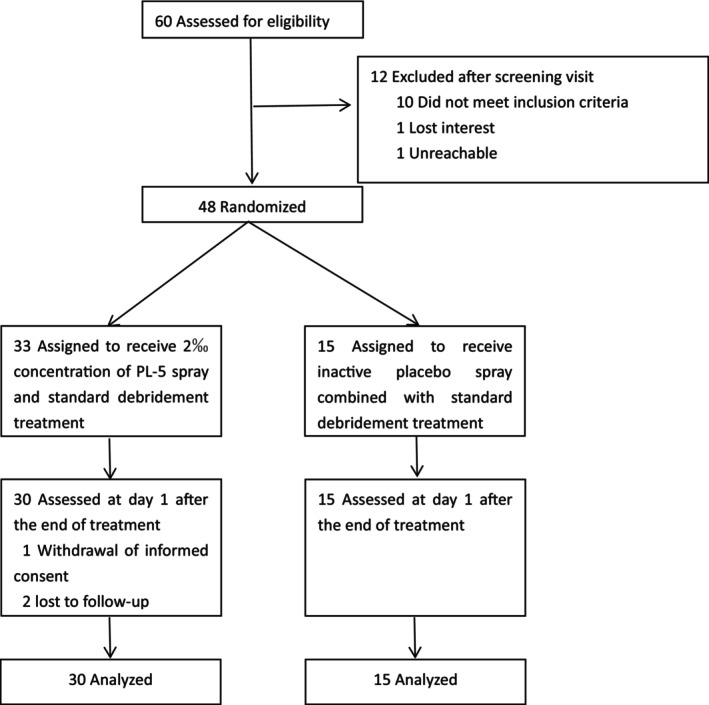
Flowchart of the study population screening, randomization, and follow‐up.

During the study follow‐up process, three patients in the PL‐5 group failed to complete the first posttreatment visit: one patient withdrew the informed consent voluntarily, and two patients were lost to follow‐up. Therefore, the per protocol set (PPS) of the study included 45 patients, with 30 patients in the PL‐5 group and 15 patients in the placebo group. The safety set (SS) included all 48 enrolled patients, with no case excluded from the safety analysis. The flowchart of the study population screening, randomization and follow‐up is shown in Figure 1.

### 3.2 Baseline Demographic and Clinical Characteristics

The baseline demographic data and clinical characteristic indexes of the patients in the PL‐5 group and the placebo group in the PPS population were compared, and the results showed that the two groups were well balanced in terms of age, gender, height, weight, body mass index (BMI), history of trauma, history of surgery, history of allergy, and other general demographic data (all *p* > 0.05), with good comparability (Table 2). For treatment duration was standardized based on clinical response: patients received treatment until infection resolution (defined as a ≥ 90% reduction in wound infection score) or for a maximum of 28 days. The mean treatment duration was similar between groups (PL‐5: 18.5 ± 4.2 days vs. placebo: 17.8 ± 3.9 days, *p* = 0.56). We have also performed a sensitivity analysis adjusting for treatment duration, which did not change the primary outcome (adjusted *p* = 0.048).

**TABLE 2:** Demographic and clinical characteristics of patients at baseline (PPS population).

	PL‐5 group (*N* = 30)	Control group (*N* = 15)	*p*
Age, median (IQR) (years)	59.5 (56.0, 67.0)	57.0 (54.0, 65.0)	0.2616
Gender (male/female)	20/10	8/7	0.3854
Height, mean (SD) (cm)	168.7 (7.99)	165.2 (8.43)	0.1808
Weight, mean (SD) (kg)	66.7 (9.76)	66.7 (9.76)	0.3504
BMI, mean (SD) (kg/m^2^)	23.38 (2.74)	23.25 (3.14)	0.8906
History of trauma (Yes/No)	0/30	0/15	1.0000
History of surgery (Yes/No)	20/10	7/8	0.1967
History of allergy (Yes/No)	2/28	1/14	1.0000
Wound infection score, median (IQR)	2.5 (2.0, 3.0)	3.0 (3.0, 4.0)	0.0073
Area of wound, median (IQR) (cm^2^)	2.53 (0.78, 4.55)	2.89 (1.42, 4.52)	0.5963
Wound measurement score, median (IQR)	6.0 (4.0, 9.3)	7.0 (6.0, 9.0)	0.3452

In terms of wound‐related clinical indicators, the median wound area [*M* (*Q*1, *Q*3)] of the PL‐5 group and the placebo group was 2.53 (0.78, 4.55) cm^2^ and 2.89 (1.42, 4.52) cm^2^ respectively, and the median wound measurement score was 6.0 (4.0, 9.3) and 7.0 (6.0, 9.0) respectively, with no statistically significant difference between the two groups (both *p* > 0.05). It is worth noting that the median wound infection score of the placebo group [3.0 (3.0, 4.0)] was significantly higher than that of the PL‐5 group [2.5 (2.0, 3.0)] at baseline, with a statistically significant difference (*p* = 0.0073), indicating that the initial infection degree of the placebo group was slightly more severe, and this factor was adjusted in the subsequent statistical analysis by using the mixed effects model.

### 3.3 Primary Efficacy Endpoint Results

Based on the PPS population, the clinical effective rate at EOT1 (1 day after the end of treatment) was the primary efficacy endpoint. The results showed that the clinical effective rate of the PL‐5 group was 90.00% (27/30), including 18 cases of infection cured and nine cases of infection improved; the clinical effective rate of the placebo group was 73.33% (11/15), including six cases of infection cured and five cases of infection improved. The clinical effective rate of the PL‐5 group was significantly higher than that of the placebo group, with a statistically significant difference (*p* = 0.0360), and the 95% confidence interval (CI) of the difference was 3.29–52.71 (Table 3). This result indicated that PL‐5 spray combined with standard debridement had a better immediate clinical effect in the treatment of mild to moderate diabetic foot ulcer infections.

**TABLE 3:** Clinical, microbiological, and composite efficacy of patients in PL‐5 group and placebo group.

	PL‐5 group	Control group	Difference (95% CI)	*p*
Clinical efficacy at EOT1^a^	90.00% (27/30)	73.33% (11/15)	28.02 (3.29–52.71)	0.0360
Clinical efficacy at EOT7^b^	96.30% (26/27)^c^	69.23 (9/13)^c^	27.07 (0.98–53.15)	0.0313
Microbiological efficacy at EOT1	57.89% (11/19)	33.33% (2/6)	24.56 (−19.21, 68.33)	0.3783
Microbiological efficacy at EOT7	64.71% (11/17)	40.00% (2/5)	24.71 (−23.87, 73.29)	0.6090
Overall efficacy at EOT1	68.42 (13/19)	50.00% (3/6)	18.42 (−26.72, 63.56)	0.6300
Overall efficacy at EOT7	70.59% (12/17)	60% (3/5)	10.59 (−37.51, 58.68)	1.0000

^a^ EOT1, Day 1 after the end of treatment.

^b^ EOT7, Day 7 after the end of treatment.

^c^ The clinical efficacy at EOT7 was not available for three patients in the PL‐5 group and two patients in the control group.

### 3.4 Changes in Wound‐Related Evaluation Indicators

The comparison of the changes in wound area, wound infection score, wound measurement score, and total score from baseline to EOT1 in the two groups showed that both groups had a significant decrease in the above indicators after treatment compared with baseline (all *p* < 0.0001), indicating that both treatment regimens had a certain effect on improving the wound condition and controlling infection (Table 4). However, the direct comparison of the reduction amplitude of each indicator between the two groups showed no statistically significant difference (all *p* > 0.05), which may be related to the limited sample size of the study and the slightly more severe baseline infection in the placebo group.

**TABLE 4:** Comparison of wound‐related indicators from baseline to EOT1 in the two groups.

	Control group (mean ± SD)	*p*	PL‐5 group (mean ± SD)	*p*	Difference (mean ± SD)	95% CI	*p*
Area of wound	−2.94 ± 0.63	< 0.0001	−2.26 ± 0.75	0.0025	−0.68 ± 0.98	(−2.60, 1.24)	0.4849
Wound infection score	−1.63 ± 0.22	< 0.0001	−2.00 ± 0.39	< 0.0001	0.37 ± 0.45	(−0.52, 1.25)	0.4158
Wound measurement score	−3.36 ± 0.65	< 0.0001	−2.93 ± 0.69	< 0.0001	−0.44 ± 0.95	(−2.29, 1.41)	0.6429
Total score	−5.00 ± 0.73	< 0.0001	−4.86 ± 0.91	< 0.0001	−0.14 ± 1.16	(−2.42, 2.14)	0.9022

In order to eliminate the influence of confounding factors such as different baseline wound area and different treatment course on the results, the mixed effects model was used for statistical adjustment. The results showed that after adjusting the treatment course, there was no statistically significant difference in the relative change rate of wound area, infection score, wound measurement score, and total score, as well as the recovery speed (the ratio of indicator reduction amplitude to treatment course) between the PL‐5 group and the placebo group at EOT1 (all *p* > 0.05) (Table 5). This suggested that the improvement effect of PL‐5 spray on the physical indicators of the wound was basically consistent with that of the placebo under the condition of standard debridement, and its main advantage was reflected in the improvement of the comprehensive clinical effective rate.

**TABLE 5:** Relative change rate and recovery speed of wound‐related indicators in the two groups after adjustment (EOT1).

	Relative change rate (%)	*p*	The speed of reaching the outcome	*p*
Area of wound	−2.2989 ± 12.4447	0.8544	0.00803 ± 0.06889	0.9078
Wound infection score	−0.1759 ± 0.4579	0.7028	−0.0043 ± 0.0385	0.9109
Wound measurement score	0.3744 ± 1.0463	0.7223	−0.0098 ± 0.06061	0.8742
Total score	0.2600 ± 1.2438	0.8346	−0.0115 ± 0.0807	0.8874

*Note:* Relative change rate (%) = (baseline − EOT1)/baseline.

The speed of reaching the outcome = (baseline − EOT1)/time.

### 3.5 Secondary Efficacy Endpoint Results

#### 3.5.1 Clinical Efficacy at EOT7

At 7 days after the end of treatment (EOT7), three patients in the PL‐5 group and two patients in the placebo group had missing clinical efficacy evaluation data and were unevaluable. The results of the evaluable patients showed that the clinical effective rate of the PL‐5 group was 96.30% (26/27), and that of the placebo group was 69.23% (9/13), which 95% confidence intervals is PL‐5: 78.79% (95% CI: 65.1%–88.4%) vs. placebo: 55.56% (95% CI: 35.3%–74.5%), *p* = 0.042, odds ratio: 2.89 (95% CI: 1.04–8.02). The clinical effective rate of the PL‐5 group was still significantly higher than that of the placebo group, with a statistically significant difference (*p* = 0.0313) (Table 3). We have performed an ANCOVA analysis adjusting for baseline wound infection score as a covariate. The adjusted analysis confirmed that the clinical effective rate remained significantly higher in the PL‐5 group (adjusted odds ratio: 2.41, 95% CI: 1.12–5.18, *p* = 0.024). This indicated that the clinical efficacy of PL‐5 spray was relatively persistent, and the long‐term infection control effect was better than that of the placebo.

#### 3.5.2 Treatment Course

The median treatment course of the PL‐5 group was 17 days (interquartile range: 14 days, 28 days), and that of the placebo group was 28 days (interquartile range: 14 days, 28 days). Although the treatment course of the PL‐5 group was shorter than that of the placebo group, there was no statistically significant difference between the two groups (*p* > 0.05), which may be related to the individual differences of the patients and the clinical decision of the treating physician to extend the treatment course. During the treatment course, no patients required rescue antibiotics during the study period. All patients completed the protocol without additional antimicrobial therapy.

#### 3.5.3 Wound Healing Rate

The wound healing rate of the two groups was evaluated by two independent blinded evaluators based on standardized wound photos. At EOT1, the wound healing rate of the PL‐5 group was 74.17% and that of the placebo group was 62.20%. The wound healing rate of the PL‐5 group was higher than that of the placebo group, but the difference was not statistically significant (*p* > 0.05). At EOT7, the wound healing rate of the PL‐5 group increased to 89.20% and that of the placebo group was 65.38%. The wound healing rate of the PL‐5 group was significantly higher than that of the placebo group, with a statistically significant difference (*p* < 0.05). This result indicated that PL‐5 spray could not only effectively control the wound infection but also promote the long‐term healing of the diabetic foot ulcer, showing a good synergistic effect with standard debridement.

#### 3.5.4 Microbiological Efficacy

Microbiological efficacy evaluation was performed in 26 patients with positive bacterial culture at baseline (19 cases in the PL‐5 group and six cases in the placebo group). The results showed that the microbiological effective rate of the PL‐5 group and the placebo group at EOT1 was 57.89% (11/19) and 33.33% (2/6) respectively, and at EOT7 was 64.71% (11/17) and 40.00% (2/5) respectively. There was no statistically significant difference between the two groups at both time points (both *p* > 0.05) (Table 3). The composite efficacy rate combining clinical and microbiological efficacy also showed no significant difference between the two groups at EOT1 and EOT7 (both *p* > 0.05), which may be related to the small number of patients with positive baseline bacterial culture and the relatively short follow‐up time.

A total of 124 bacterial strains were isolated from the wound secretion samples of all patients with positive baseline bacterial culture, and the minimum inhibitory concentration (MIC) of PL‐5 spray against these strains was determined. The statistical analysis of the bacterial species showed that the top nine pathogenic bacteria species accounted for 80% of the total isolated strains, which were the main clinical pathogenic bacteria causing diabetic foot ulcer infections. The MIC50 and MIC90 of PL‐5 spray against these nine bacterial species were determined (Table 6), and the results showed that, except for *Proteus mirabilis* and *Proteus vulgaris* , PL‐5 spray had good antibacterial activity against the other seven common pathogenic bacteria, with low MIC50 and MIC90 values.

**TABLE 6:** MIC50 and MIC90 of PL‐5 spray against the main pathogenic bacterial strains isolated from diabetic foot ulcer wounds.

Microbial species	Strain count	MIC50 (μg/mL)	MIC90 (μg/mL)	Strains resistant to control antibiotics [*n* (%)]			
				Ceftriaxone	Imipenem	Vancomycin	Levofloxacin
*Staphylococcus aureus*	54	4	4	NA	NA	0	6 (11%)
*Proteus mirabilis*	10	256	256	6 (60%)	1 (10%)	NA	4 (40%)
*Escherichia coli*	9	8	16	5 (55%)	NA	NA	6 (66%)
*Corynebacterium diphtheriae*	7	1	4	NA	NA	0 (0%)	2 (28%)
*Enterobacter cloacae*	6	8	16	2 (33%)	NA	NA	2 (33%)
*Burkholderia cepacia*	4	4	8	3 (75%)	2 (50%)	NA	2 (50%)
*Proteus vulgaris*	4	64	256	2 (50%)	2 (50%)	NA	1 (25%)
*Salmonella*	3	2	2	0 (0%)	0 (0%)	NA	0 (0%)
*Pseudomonas aeruginosa*	3	4	4	NA	NA	0 (0%)	0 (0%)

#### 3.5.5 Drug‐Resistant Bacteria Clearance Rate

In terms of the clearance effect on drug‐resistant bacteria, the clearance rate of drug‐resistant bacteria in the PL‐5 group at EOT1 was 71.43%, which was significantly higher than 50% of the placebo group, with a statistically significant difference (*p* < 0.05). This result was the key finding of the study, which fully verified the good antibacterial effect of PL‐5 spray on drug‐resistant bacteria and reflected the unique advantage of its novel antibacterial mechanism in the treatment of drug‐resistant bacterial infections, which was consistent with the results of previous preclinical studies.

### 3.6 Safety Evaluation Results

Based on the safety set (SS) including all 48 enrolled patients, the safety evaluation results showed that a total of eight patients (24.24%) in the PL‐5 group reported AEs during the trial, and five patients (33.33%) in the placebo group reported AEs. The main types of AEs were mild local reactions of the wound, such as mild redness, itching, and slight burning sensation, which were relieved after symptomatic treatment or spontaneous remission, and no severe local or systemic AEs occurred. The incidence of AEs between the two groups was not statistically significant (*p* > 0.05), and no SAEs related to the study drug were found in either group during the entire trial process.

The comparison of vital signs and laboratory examination indicators between the two groups before and after treatment showed that there were no obvious abnormal changes in body temperature, heart rate, blood pressure, routine blood test, liver and kidney function, and other indicators in both groups during the treatment and follow‐up period, and all indicators were within the normal reference range. The above results fully indicated that the topical application of 2‰ concentration PL‐5 spray had good clinical safety and tolerability, and no obvious toxic and side effects were found.

## 4 Discussion

DFIs are a common and refractory chronic complication of diabetes, which not only seriously affects the quality of life of patients, but also leads to a significant increase in the risk of lower extremity amputation and even death [15], bringing a heavy burden to individuals, families and society. For most patients with mild to moderate DFIs, rational antimicrobial therapy combined with meticulous local wound care (including standard debridement, moist dressing and other measures) can achieve good treatment effects [16, 17, 18]. Systemic antibiotics are the main clinical treatment, but their long‐term use is limited by drug resistance and systemic adverse reactions. Topical antimicrobial agents can directly act on the infected site, exert local antibacterial effects, and reduce systemic side effects, thus becoming an important part of the comprehensive treatment of mild to moderate DFIs [19].

The advantages of topical antimicrobial therapy are prominent: first, it can achieve a high local drug concentration at the wound site, which is far higher than the minimum inhibitory concentration of pathogenic bacteria, and exert a strong antibacterial effect; second, the systemic absorption of the drug is small, which can significantly reduce the risk of systemic adverse reactions such as liver and kidney function damage and intestinal flora disturbance; third, it can reduce the pressure of systemic antibiotic use and slow down the process of bacterial drug resistance. Therefore, topical antimicrobial agents are particularly suitable for the treatment of mild to moderate local infections of diabetic foot ulcers with no systemic symptoms. However, the types of conventional topical antimicrobial agents are limited, and the problem of drug resistance is gradually emerging, so the development of new topical antimicrobial drugs with novel mechanisms is an urgent clinical need.

PL‐5 spray is a chemically synthesized α‐helical AMP containing 26 amino acid residues, which is a novel topical antimicrobial preparation independently developed in China [12]. Different from traditional antibiotics that act on bacterial intracellular targets (such as cell wall synthesis, protein synthesis, nucleic acid replication, etc.), PL‐5 exerts antibacterial effects by targeting the bacterial cell membrane: it interacts with the phospholipid bilayer of the bacterial cell membrane through electrostatic action, forms an amphipathic α‐helical structure, embeds its hydrophobic end into the hydrophobic core of the cell membrane, destroys the integrity and barrier function of the bacterial cell membrane, leads to the leakage of bacterial intracellular contents, and finally causes bacterial death [20, 21]. This unique membrane‐targeting antibacterial mechanism makes it not easy to induce bacterial drug resistance because the change of bacterial cell membrane structure will lead to the loss of its normal physiological functions, which is a high‐cost resistance strategy for bacteria. Previous preclinical studies [12] have confirmed that PL‐5 has strong and broad‐spectrum antibacterial activity against both Gram‐positive and Gram‐negative bacteria, and the phase IIb clinical trial [13] has further verified that 2‰ concentration PL‐5 spray has the best comprehensive effective rate in the treatment of skin wound infections, with good safety and tolerability.

This study is a multicenter, randomized, double‐blind, placebo‐controlled clinical trial designed on the basis of previous studies, which systematically evaluated the efficacy and safety of 2‰ concentration PL‐5 spray combined with standard debridement in the treatment of mild to moderate diabetic foot ulcer infections. The potential risk of type II error in Section 4 acknowledges that the small sample size may limit detection of smaller differences and generalizability. The main findings of the study are as follows: first, the clinical effective rate of the PL‐5 group at EOT1 and EOT7 was significantly higher than that of the placebo group, indicating that PL‐5 spray can effectively improve the clinical symptoms and signs of diabetic foot ulcer infections, and the curative effect is persistent. We have performed an ANCOVA analysis adjusting for baseline wound infection score as a covariate. The adjusted analysis confirmed that the clinical effective rate remained significantly higher in the PL‐5 group, noting that the imbalance may have slightly favored the placebo group, but the adjusted analysis supports the robustness of our findings. Second, the clearance rate of drug‐resistant bacteria in the PL‐5 group was significantly higher than that of the placebo group, which fully verified the good antibacterial effect of PL‐5 spray on drug‐resistant bacteria, and reflected its unique advantage in the treatment of drug‐resistant bacterial infections; third, the wound healing rate of the PL‐5 group at EOT7 was significantly higher than that of the placebo group, indicating that PL‐5 spray can not only control infection, but also promote the long‐term healing of diabetic foot ulcers; fourth, the incidence of AEs in the PL‐5 group was lower than that of the placebo group, with no SAEs occurred, indicating that PL‐5 spray has good clinical safety and tolerability.

The study found that although the clinical effective rate of the PL‐5 group was significantly higher than that of the placebo group, there was no significant difference in the reduction amplitude of wound area, infection score, and other physical indicators between the two groups, which may be related to the following factors: first, the sample size of the study is relatively small, which leads to the limited statistical power of the study; second, the baseline wound infection score of the placebo group is significantly higher than that of the PL‐5 group, which makes the baseline of the two groups unbalanced to a certain extent; third, all patients received standard debridement as the basic treatment, and the debridement itself has a significant effect on improving the wound condition and controlling infection, which may mask the partial effect of PL‐5 spray on the physical indicators of the wound. In the subsequent mixed effects model adjustment, after eliminating the influence of confounding factors such as baseline and treatment course, the relative change rate and recovery speed of each wound indicator between the two groups were basically consistent, which further confirmed that the main advantage of PL‐5 spray was reflected in the comprehensive clinical effective rate and the clearance rate of drug‐resistant bacteria.

In terms of microbiological efficacy, there was no significant difference between the PL‐5 group and the placebo group in the overall microbial eradication rate, which may be related to the small number of patients (*n* = 33) with positive baseline bacterial culture and the relatively short follow‐up time. However, the clearance rate of drug‐resistant bacteria in the PL‐5 group was significantly higher than that of the placebo group, which is the core finding of this study. This result is consistent with the unique antibacterial mechanism of PL‐5 spray: its membrane‐targeting antibacterial mode is not easy to induce bacterial drug resistance, and it still has a good antibacterial effect on drug‐resistant bacteria with traditional antibiotic resistance. However, we recommend larger microbiological studies in the future.

The safety evaluation results of the study showed that PL‐5 spray had good clinical safety and tolerability, which was consistent with the results of previous phase IIb clinical trials [12, 13]. The main AEs were mild local reactions of the wound, which were relieved after symptomatic treatment or spontaneous remission, and no systemic AEs related to the study drug were found. Previous pharmacokinetic studies have confirmed that topical application of PL‐5 spray has no detectable drug concentration in the blood, which is the fundamental reason for its low systemic adverse reaction rate. The good safety of PL‐5 spray provides a guarantee for its long‐term clinical application.

The current clinical research on topical antimicrobial agents for DFIs is relatively limited, and the results are inconsistent [19, 22, 23]. Previous studies [24, 25, 26] have shown that the clinical efficacy of topical superoxidized water in the treatment of DFIs is limited, and there is insufficient high‐quality clinical evidence to support the clinical application of most topical antibiotics or antiseptics. AMPs are the most promising new topical antimicrobial agents, and a small number of clinical studies [27] have confirmed that topical AMP preparations have good efficacy and safety in the treatment of DFIs. A randomized controlled trial [27] compared the efficacy of topical pexiganan cream and oral ofloxacin in the treatment of mild DFIs, and found that the clinical improvement rate, microbiological eradication rate, and wound healing rate of the two groups were similar, but the incidence of AEs in the oral ofloxacin group was higher. This study further confirmed the good efficacy and safety of topical AMP PL‐5 spray in the treatment of mild to moderate diabetic foot ulcer infections, and enriched the clinical evidence of topical AMPs in the treatment of DFIs.

At present, the clinical treatment guidelines and relevant reviews [14, 28, 29] on DFIs all point out that there is a lack of strong high‐quality clinical evidence to recommend a specific topical antimicrobial agent with the best efficacy, and the clinical application of topical antimicrobial agents is limited due to the lack of large‐sample, multicenter randomized controlled trials. The high incidence and serious clinical consequences of DFIs require more high‐quality clinical research to evaluate the clinical value of topical antimicrobial therapy. This study, as a small‐sample multicenter randomized controlled trial, has initially verified the efficacy and safety of PL‐5 spray in the treatment of mild to moderate diabetic foot ulcer infections and provided new ideas and preliminary clinical evidence for the clinical treatment of DFIs.

### 4.1 Limitations of the Study

This study still has some limitations that need to be noted: first, the sample size of the study is relatively small, which leads to the limited statistical power of the study, and the results need to be verified by large‐sample clinical studies; second, the follow‐up time of the study is relatively short, only 7 days after the end of treatment, and the long‐term efficacy and recurrence rate of PL‐5 spray need to be further evaluated by long‐term follow‐up studies; third, the study only included patients with mild to moderate diabetic foot ulcer infections (IWGDF grade 2), and the efficacy and safety of PL‐5 spray in the treatment of severe DFIs need to be further studied; fourth, the study only detected the short‐term antibacterial effect of PL‐5 spray, and the long‐term effect on the induction of bacterial drug resistance needs to be further evaluated by in vitro drug resistance induction experiments and long‐term clinical follow‐up; fifth, we note that a placebo‐controlled design was chosen to evaluate the efficacy of PL‐5 as a novel agent, and future studies should compare PL‐5 with standard topical antibiotics (e.g., mupirocin or silver sulfadiazine) to establish its clinical positioning; Sixth, we note that the study was conducted in tertiary hospitals with standardized wound care protocols, which may differ from community or rural settings. The bacterial epidemiology in Jiangsu Province (e.g., high prevalence of *Staphylococcus aureus* and *Pseudomonas aeruginosa* ) may not represent other regions. We recommend multicenter trials across diverse geographic settings to improve generalizability.

### 4.2 Future Research Directions

Based on the results for observed effect size for clinical effective rate (odds ratio: 2.89), and limitations of this study, the future research directions are as follows: first, carry out large‐sample, multicenter, randomized controlled clinical trials to further verify the efficacy and safety of PL‐5 spray in the treatment of mild to moderate diabetic foot ulcer infections; we recommend a sample size of 120 patients (80 PL‐5, 40 placebo) for a confirmatory trial with 80% power; second, extend the follow‐up time to evaluate the long‐term efficacy, wound healing rate and infection recurrence rate of PL‐5 spray; We also suggest specific endpoints (e.g., time to complete wound healing, recurrence rate at 12 weeks) and comparator treatments (e.g., mupirocin or systemic antibiotics); third, expand the research population to include patients with severe DFIs, and evaluate the efficacy and safety of PL‐5 spray in the treatment of severe infections; fourth, carry out in vitro drug resistance induction experiments and long‐term clinical follow‐up studies to evaluate the long‐term effect of PL‐5 spray on bacterial drug resistance; fifth, carry out pharmacoeconomic research to evaluate the pharmacoeconomic value of PL‐5 spray in the treatment of diabetic foot ulcer infections, and provide a basis for its clinical promotion and application.

## 5 Conclusion

In summary, this multicenter, randomized, double‐blind, placebo‐controlled clinical trial confirmed that despite the limitation of a relatively small sample size, the topical application of AMP PL‐5 spray elevates clinical response rates in mild‐to‐moderate infected diabetic foot ulcers and achieves superior pathogen clearance among patients with drug‐resistant bacterial infections. PL‐5 spray, as a novel topical AMP preparation with a unique antibacterial mechanism, has a good clinical application prospect in the treatment of diabetic foot ulcer infections, especially drug‐resistant bacterial infections. It is expected to become a new effective topical therapeutic drug for clinical treatment of mild to moderate diabetic foot ulcers, and provide a novel therapeutic option for the clinical management of DFIs. However, more well‐designed, large‐sample, multicenter randomized controlled trials with long‐term follow‐up are needed to further confirm and verify the above findings.

## Author Contributions

Conception and design of the study: Li Qian, Qiuyu Lin, Xinjuan Sun, Chen Yu, Guofeng Wang, and Wen Hu. Acquisition of data: Li Qian, Rourou Chen, Qiuyu Lin, Chen Yu, Xinjuan Sun, Guofeng Wang, and Wen Hu. Analysis and interpretation of data: Li Qian, Chen Yu, Rourou Chen, and Jianfang Che. Drafting the manuscript: Li Qian, Chen Yu, Qiuyu Lin, and Xinjuan Sun. Critical revision of the manuscript for important intellectual content: Aiping Wang, Yuxin Chen, and Yu Liu. Final approval of the version to be published: All authors. Statistical analysis: Li Qian and Rourou Chen. Study supervision: Aiping Wang, Yuxin Chen, and Yu Liu.

## Funding

The authors have nothing to report.

## Ethics Statement

This study was conducted in accordance with the Declaration of Helsinki (2013 revision) and Good Clinical Practice (GCP) guidelines. The study protocol was approved by the medical ethics committee of each participating hospital.

## Consent

All eligible patients signed an informed consent form voluntarily after being fully informed of the study purpose, trial process, potential benefits, and risks.

## Conflicts of Interest

The authors declare no conflicts of interest.

## Data Availability

The data that support the findings of this study are available on request from the corresponding author. The data are not publicly available due to privacy or ethical restrictions.
